# Rapid analysis method for the determination of ^14^C specific activity in irradiated graphite

**DOI:** 10.1371/journal.pone.0191677

**Published:** 2018-01-25

**Authors:** Vidmantas Remeikis, Elena Lagzdina, Andrius Garbaras, Arūnas Gudelis, Jevgenij Garankin, Rita Plukienė, Laurynas Juodis, Grigorijus Duškesas, Danielius Lingis, Vladimir Abdulajev, Artūras Plukis

**Affiliations:** Center for Physical Sciences and Technology, Vilnius, Lithuania; University of Liverpool, UNITED KINGDOM

## Abstract

^14^C is one of the limiting radionuclides used in the categorization of radioactive graphite waste; this categorization is crucial in selecting the appropriate graphite treatment/disposal method. We propose a rapid analysis method for ^14^C specific activity determination in small graphite samples in the 1–100 μg range. The method applies an oxidation procedure to the sample, which extracts ^14^C from the different carbonaceous matrices in a controlled manner. Because this method enables fast online measurement and ^14^C specific activity evaluation, it can be especially useful for characterizing ^14^C in irradiated graphite when dismantling graphite moderator and reflector parts, or when sorting radioactive graphite waste from decommissioned nuclear power plants. The proposed rapid method is based on graphite combustion and the subsequent measurement of both CO_2_ and ^14^C, using a commercial elemental analyser and the semiconductor detector, respectively. The method was verified using the liquid scintillation counting (LSC) technique. The uncertainty of this rapid method is within the acceptable range for radioactive waste characterization purposes. The ^14^C specific activity determination procedure proposed in this study takes approximately ten minutes, comparing favorably to the more complicated and time consuming LSC method. This method can be potentially used to radiologically characterize radioactive waste or used in biomedical applications when dealing with the specific activity determination of ^14^C in the sample.

## Introduction

Graphite has been used as a moderator and reflector material in nuclear reactors (Chicago Pile-1 (USA), UNGG (Natural Uranium Graphite Gas reactor, France), Magnox (United Kingdom) and RBMK (High Power Channel-type Reactor, former Soviet Union) since the very beginning of nuclear energy development, and considerable amounts of this radioactive material have been accumulated worldwide [1]. Due to its low neutron capture and high scattering cross section, as well as its high chemical and thermal stability, graphite is the intended for use in some of the newly designed generation IV reactors (VHTR (Very High Temperature Reactor), MSR (Molten-Salt Reactor). Many of the old reactors are shut down and their decommissioning is in progress. Irradiated graphite from the reactor core, moderators and reflectors represents the greatest volume of waste materials [2,3]. Graphite waste can be disposed of in the near surface or in a deep geological repository depending on its specific activity [4].

The ^14^C isotope is one of the greatest concerns in the long-term disposal of irradiated graphite due to its long half-life (5700 (30) years) and relatively high specific activity in graphite, as well as its mobility in geological media. ^14^C is generated in reactor graphite due to the neutron capture by ^13^C located in the graphite matrix or by the neutron activation of ^14^N and ^17^O impurities. The main contributor to ^14^C generation in the RBMK reactor is the ^14^N(n, p)^14^C reaction. This occurs mostly on the graphite surface because of the sufficient isotopic abundance of ^14^N due to presence of a helium-nitrogen mixture that is used to flush the graphite stack in the core, as well as the high neutron reaction cross section [2,3,5]. Assuming that a considerable part of ^14^C is located in the outer graphite layer, removal of ^14^C from the graphite surface would allow decreasing the total activity of graphite. Several methods have been proposed to separate ^14^C from bulk carbon: thermal treatment with steam [1], bioseparation [6], combustion [7], heating under Ar atmosphere [8], and so forth. Incineration of 1700 tons of graphite from one RBMK-1500 reactor would result in the release of ~7 × 10^14^ Bq of ^14^C, increasing the amount of ^14^C in the atmosphere by 0.6% [9]. The prevailing contamination product removed during the thermal treatment of irradiated graphite has previously been reported as ^14^C in the form of ^14^CO or ^14^CO_2_, but the detail analysis of the chemical form of ^14^C within the graphite matrix was not investigated. Characterization of the chemical nature of ^14^C in irradiated graphite may enable the optimization and development of the chemical and physical processes to remove outer graphitic layers [10,11].

The ^14^C specific activity in RBMK irradiated graphite is about 10^5^ Bq/g for the graphite within the reactor central part of the core and can be one order of magnitude lower in the graphite reflectors, where the neutron flux is also an order of magnitude lower [12]. These concentration values are considerably higher than the exemption level, equal to 1 Bq/g, for ^14^C in bulk materials [13]. Similarly, the specific activity limit for a near surface vault disposal system is far lower than these concentrations (limit of ~8 × 10^3^ Bq/g) [14]. The specific activity of RBMK radioactive graphite waste should be reduced by at least one order of magnitude and more to fit for the surface disposal. It reveals the potential benefits of treating the graphite before its disposal: if one could apply a specific graphite treatment procedure to reduce the average specific activity of ^14^C and some other nuclides to meet the acceptance criteria, the disposal of irradiated graphite in a near surface repository, instead of costly deep geological repository, could be possible. This would de-categorize the graphite from intermediate activity long-lived waste to low activity long-lived radioactive waste and make this material less problematic.

In order to develop feasible graphite treatment techniques, it is indispensable to apply effective characterization methods which can provide fast activity analyses of graphite samples. Moreover, the specific activity of graphite samples is important if one aims to compare measurement results with waste acceptance criteria or other specific limits. In this paper, we concentrate on the determination of ^14^C activity in irradiated graphite samples, which is one of the limiting radionuclides in radioactive waste level determination. As ^14^C is a β-ray emitter, radiochemical separation is necessary prior to the measurement procedure. For this task, several techniques can be applied. The first method includes sample decomposition using a mixture of inorganic acids to obtain a clear solution followed by measurement using the LSC (Liquid Scintillation Counter) technique [15]. Another method involves combustion in pure oxygen at a high temperature (600–1000°C) to convert the carbon into a gaseous form. If necessary, CO is further converted to CO_2_ which is then collected by trapping in an alkali solution. The measurement of ^14^C activity is then achieved using LSC or gas activity detectors. The LSC method is effective but due to the long time required to obtain results, it cannot be easily used in the case when a prompt analysis of a large number of samples is necessary.

For practical applications in the nuclear industry and other fields (such as biomedicine) it is important to be able to measure ^14^C activity in small samples, and prompt analysis would also be an advantage. The aim of this work is to provide the basis for a rapid analysis method that determines ^14^C specific activity in small (less than 100 μg) samples of carbonaceous materials. This method can be applied for the prompt, online, radiological characterization of irradiated graphite during dismantling of reactor graphite constructions and during the sorting of graphite waste, as well as in measuring ^14^C concentration in other (for example, bioorganic) materials.

## Experimental equipment and analysis methods

Graphite samples were obtained from the Ignalina NPP RBMK-1500 reactor, in which graphite was used as both a moderator and a reflector. The samples were collected from different reactor sites—from central and peripheral zones. Samples No. 3–5 and No. 8–10 were selected from the central zone of the reactor, while samples No. 1 and No. 2 were from the peripheral zone. Some of the samples were divided into several parts for analysis.

Prior to combustion, the mass of each graphite sample was determined using a XP105 (Mettler-Toledo, Switzerland) dual range balance. The results are listed in Table 1, organized as the sample mass by weighing. The amount of carbon in the CO_2_ form that evolved during the combustion process was estimated using a thermal conductivity detector TCD from an elemental analyzer.

**Table 1:** Experimentally determined activity of ^137^Cs, ^60^Co and ^14^C in the graphite sample. The mass of the same sample was determined by two methods: weighing with balances, and later by combustion in an elemental analyzer.

Sample No.	Activity, Bq			^14^C specific activity (by CO_2_ LSC), Bq/g	Activity ratio ^14^C/^60^Co	Sample mass, μg	
	^137^Cs	^60^Co	^14^C				
						by weighing	by CO_2_ amount
1	-	0.09 ± 0.03	16.9 ± 1.1	(2.4 ± 0.2) × 10^5^	188 ± 49	70±5	72±3
2	-	0.11 ± 0.03	21.4 ± 1.3	(1.0 ± 0.6) × 10^5^	195 ± 41	214±3	209±7
3	0.04 ± 0.01	1.6 ± 0.2	198 ± 12	(7.0 ± 0.4) × 10^5^	124 ± 16	290±3	283±9
4	0.04 ± 0.01	1.4 ± 0.2	176 ± 11	(7.0 ± 0.4) × 10^5^	125 ± 16	254±3	250±9
5	-	1.1 ± 0.1	121 ± 8	(6.8 ± 0.5) × 10^5^	109 ± 14	178±4	179±6
6	-	0.40 ± 0.06	95.4 ± 5.6	(3.9 ± 0.2) × 10^5^	239 ± 34	242±3	247±8
7	-	0.64 ± 0.08	16.2 ± 1.1	(9.3 ± 0.6) × 10^4^	25 ± 4	184±4	174±6
8	0.04 ± 0.01	0.15 ± 0.03	37.5 ± 2.2	(5.1 ± 0.3) × 10^5^	250 ± 50	108±4	74±3
9		0.067 ± 0.010	12.6 ± 0.7		188 ± 39	18 ± 4	23±2
10		0.040 ± 0.007	0.16 ± 0.02		4 ± 0.8	n.a.	0.9±1

**Fig 1:**
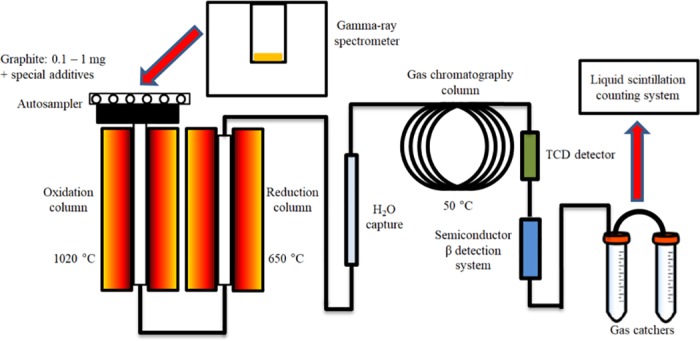
Rapid system for ^14^C specific activity determination in the sample. System contains elemental analyzer (autosampler, oxidation and reduction columns, water trap, gas chromatography column, thermal conductivity detector (TCD)) and β particle detection system. In the present study, gas catchers (3 M NaOH) and LSC were used to evaluate the accuracy of the measurements of samples with low ^14^C activity.

The experimental equipment used in this research is shown in Fig 1. It consists of both a commercial elemental analyzer, in which the graphite samples are combusted, and the mass of carbon in the sample is determined; and a semiconductor β detection system for online ^14^C determination, consisting of a chamber and 2 semiconductor detectors. Additionally, gas catchers and a liquid scintillation counting system were used to evaluate the accuracy of the semiconductor system.

### Graphite combustion and determination of CO_2_ amounts with TCD

Graphite samples were combusted using an elemental analyzer Thermo Flash EA 1112 (*Thermo Fisher*, *USA*). The elemental analyzer consists of oxidation and reduction columns (operated at 1020°C and 650°C, respectively), a water trap, a chromatographic column PoraPlot Q (*Agilent*, *USA*) (3 m long, operated at 50°C) and a thermal conductivity detector. The oxidation reactor is filled with chromium oxide and silvered cobaltous/cobaltic oxide, while the reduction reactor consists of reduced copper. The purpose of silvered cobaltous/cobaltic oxide is to trap halides (for instance, ^36^Cl which is weak beta emitter and can cause interference in ^14^C measurement, within the online detection system). Prior to the analysis, non-ground graphite samples were weighed with balances and packed into tin capsules. As it is difficult to burn down the graphite completely [16], magnesium perchlorate was added to the tin capsule together with the graphite sample as an additional oxidant. The quantity of added oxidant was 5 times larger compared to that of the graphite sample. The magnesium perchlorate in the elemental analyzer decomposes at high temperatures producing oxygen as shown [17]:

$$2Mg{\left(ClO_{4}\right)}_{2}\to [MgO∙Mg{\left(ClO_{4}\right)}_{2}]+{Cl}_{2}+{3.5O}_{2},$$ (1)

$$[MgO∙Mg{\left(ClO_{4}\right)}_{2}]\to 2MgO+{Cl}_{2}+{3.5O}_{2},$$ (2)

During the analysis, the graphite sample and an oxidant were placed into the autosampler. The autosampler was flushed with He gas at a flow rate of 180 ml/min. The sample was later dropped into the oxidation column with a helium flow rate of 80 ml/min and an excess of oxygen. The oxygen dosing time was 10 seconds with a flow rate of 80 ml/min. After the combustion event, evolved gasses passed through the reduction column, a water trap and the chromatographic column. The amount of evolved CO_2_ gas (or mass of the carbon in the sample) in the He flow was estimated using TCD. Later all gasses were passed, via a specially constructed tubing system, through the β particle registration system and sequentially to the two CO_2_ traps, composed of 3 M NaOH solution. The time interval from the start of the sample combustion to the ^14^C detection in the evolved CO_2_ gas was 400 s.

The calibration of the TCD was performed using materials with known amounts of carbon—nicotinamide and atrazine which were obtained from Thermo Electron Corporation (Italy). The standard weight pressed tin capsules with dimensions of 5 mm × 3.5 mm were obtained from Sercon Ltd (UK). High purity He 5.0 and O_2_ 4.5 gas were used during the analysis.

### ^14^C online detection in gas flow

**Fig 2:**
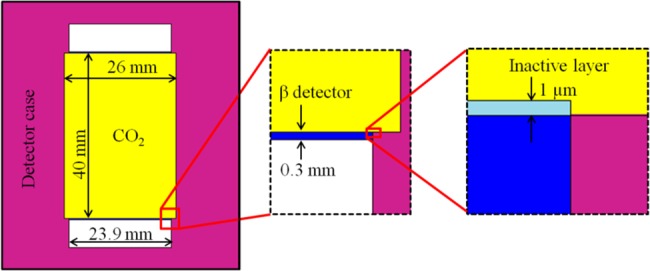
MCNP model of a detection chamber with zoomed fragments of the Si detector and inactive layer on the top of the detector.

For the purpose of β particle registration in CO_2_ gas flow, semiconductor silicon planar PIPS (Passivated Implanted Planar Silicon, Canberra, USA) detectors were used. The detecting system was located between the sample combustion system (elemental analyzer) and the CO_2_ alkali condenser, as shown in Fig 1. This type of detector was chosen because of its wide operating temperature range, ability to operate in harsh and ambient light environments, and higher efficiency as compared to other types of semiconductor detectors [18]. The active thickness of the detector was 300 μm at the depletion voltage of 70 V, and the active area was 450 mm^2^. The detection chamber diameter was 26 mm, the height was 40 mm, giving a total volume of 21.2 cm^3^. Evaluation of the ^14^C β particle detection efficiency was performed using a Monte Carlo (MCNP6) simulation [19]. The modelling accuracy was checked using a Monte Carlo simulation of the experimental measurements of ^14^C, ^137^Cs and ^90^Sr planar sources. The modelled system consisted of 2 Si semiconductor detectors each with a diameter of 23.9 mm and a height of 0.3 mm with a thin inactive layer (1 μm thick) on the top (see Fig 2). The efficiency is evaluated using the F8 tally, which gives the energy distribution of pulses created in the detector. The estimated efficiency of the system using both detectors was 15%. This low efficiency is due to the less than the ideal 2π geometry—the prolonged chamber allows a larger measurement volume.

**Fig 3:**
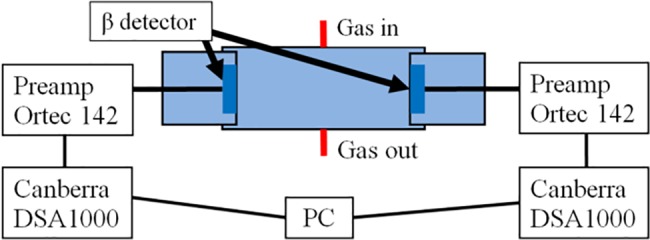
β particle registration system in CO_2_ gas flow with semiconductor detectors.

During the measurement process, gas from the sample combustion system passed through the detection chamber with two silicon detectors, where the gas activity was registered. Pulses from each of the detectors were amplified with an Ortec 142 (*Ortec*, *USA*) preamplifier and registered using a DSA1000 (Canberra, USA) system (see Fig 3).

**Fig 4:**
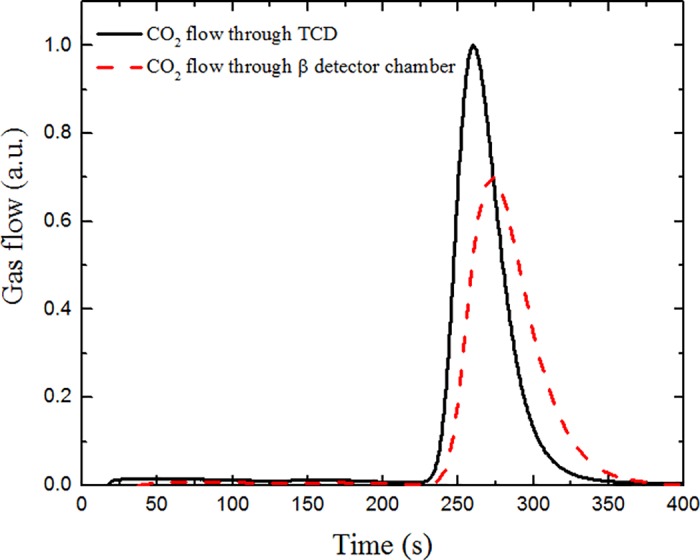
CO_2_ gas flow in the TCD and the β counter chamber during the first cycle of combustion of sample No. 6.

The bias voltage for each detector was set to 70 V, the amplifier gain was set to 100 and the pulse shape time was 2 μs. These parameters proved to be most suitable, as they resulted in the least observed noise. The LLD threshold for DSA was set to the first 10 channels during measurement to avoid a false signal due to electronic noise. Spectra were measured with a time step interval of 100 seconds, and each spectrum was integrated to find out the sum of pulses per step. ^14^C activity was measured using 4 steps per combustion cycle. During the first 2 steps only the background spectra were collected, while during the last 2 steps, CO_2_ gas flow was observed and decay particles were registered (see Fig 4). During the combustion of sample No. 6, almost all the gas (90%) flows through TCD during the third step of the first combustion cycle and only a small partition of gas flows during the start of the fourth step (10%). However, the gas flow through the semiconductor detector system is delayed and spans both the third (77%) and fourth (23%) steps.

The gas flow delay in the detection chamber occurs because the chamber volume is much larger than that of the gas tubes. Each combustion cycle does not influence the next one because simulation shows that all of the CO_2_ gas is flushed away from the chamber during the combustion cycle spanning 400 seconds. Condensation of the material is minimized as the temperature differences between the gas in the chamber and the chamber walls are controlled.

### Radiocarbon destructive dnalysis and LSC

**Fig 5:**
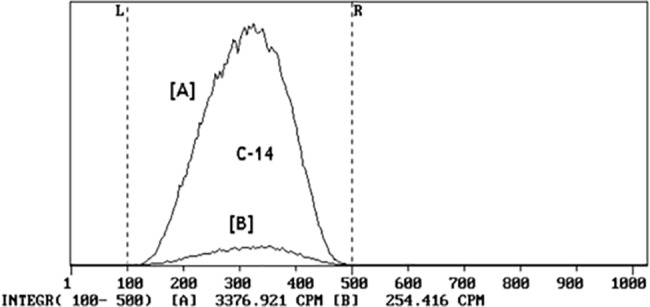
Radiocarbon spectra of a graphite sample. (**a**) from the first catcher, (**b**) from the second catcher.

After graphite combustion, the evolved CO_2_ gas was passed through the NaOH 3M solution placed in two catchers connected serially. It was known from previous experiments that the combination of two catchers ensures 94% trapping (recovery) efficiency for radiocarbon in the ^14^CO_2_ form [20]. The exposed solutions were mixed with a liquid scintillation cocktail OptiPhase HiSafe 3 (PerkinElmer, USA) applying the mixing ratio of 4 and 16 ml. Then samples were then measured with a liquid scintillation counter Quantulus-1220 (*PerkinElmer*, *USA*). LSC measurements were traceable to the national standard of activity [21]. The obtained LSC spectra confirmed the presence of pure ^14^C β activity in the samples showing no other radionuclides in the exposed NaOH 3M solution. Typical LSC spectra as measured in the samples taken from the two catchers are presented in Fig 5.

The radiocarbon activity in a graphite sample was calculated taking into account activities determined in catchers, the counting efficiency, the recovery efficiency and the aliquot volume. The standard uncertainty of the radiocarbon activity determination was around 6%.

### Gamma-ray spectrometry

Gamma-ray spectrometry was applied to each graphite sample before destructive analysis for the initial sample characterization (determination of gamma emitters and their activity). The total sample activity often correlates with ^60^Co activity and, according to ^60^Co gamma measurement, one could determine the scaling factors for other activation products (β or α emitters of the same origin) [22].

Graphite samples were measured using a gamma-ray spectrometer equipped with a HPGe well-type detector with a crystal volume of 170 cm^3^, and an energy resolution of 2.05 keV at a full-width at the half peak maximum (FWHM) of 1332.5 keV. Quality control procedures for this detector and the results from proficiency test schemes are previously described [23]. Certified reference radionuclide solutions traceable to the national standard of activity were used for efficiency calibration [24]. True coincidence-summing corrections were applied to the ^60^Co activity measurement.

## Results and discussion

### Graphite mass and activity measurements by conventional methods

The analytical results of activity measurements of gamma-ray emitters and ^14^C in graphite samples, as well as sample masses, are summarized in Table 1.

**Fig 6:**
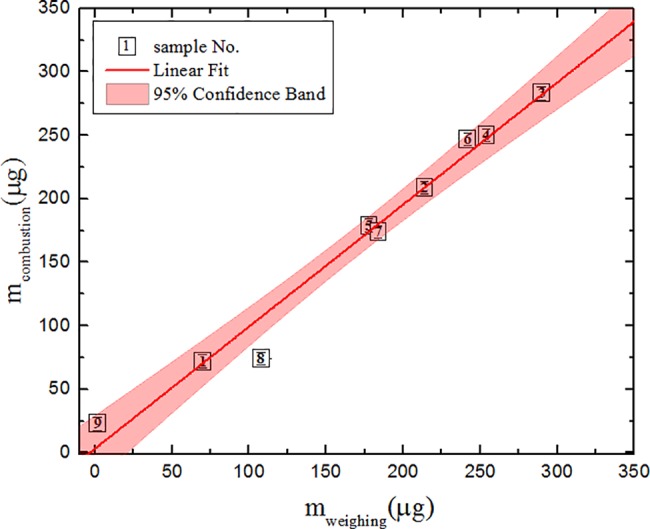
The correlation of the graphite sample mass as determined by two independent methods: Weighing and combustion in the elemental analyzer. Graphite sample mass in the same sample was determined by weighing with balances (horizontal axis) and by combustion in the elemental analyzer, measured from the resulting CO_2_ (vertical axis). The parameters of linear approximation (y = a + bx) are: a = 3 ± 10, b = 0.96 ± 0.06, R = 0.97.

The correlation of the graphite sample mass as determined by weighing and by the method used in this research is presented in Fig 6. It is evident that all measured samples (except for sample No. 8) are within the 95% confidence band and the correlation coefficient is ~0.97. It has to be noted that the proposed method is particularly useful for the prompt determination of ^14^C specific activity in small samples (less than 100 μg) making it potentially applicable not only to high activity irradiated graphite samples but also in other fields such as biomedicine.

The correlation coefficient R is determined by the expression:

$$R=\frac{n\sum xy-\sum x\sum y}{\sqrt{\left[n\sum x^{2}-{\left(\sum x\right)}^{2}][n\sum y^{2}-{\left(y\right)}^{2}\right]}},$$ (3)

where *n* is the number of samples, *x* and *y* are values of variables. If the correlation coefficient is higher than 0.8 the correlation is generally considered to be strong. A correlation coefficient below 0.5 is not acceptable for estimating the activities of difficult-to-measure radionuclides based on the activity of a key radionuclide. Using confidence bands it is possible to obtain uncertainty of the scaling factor and from the upper prediction band one can find out the upper limit of the nuclide activity in the radioactive waste for a given key nuclide activity.

**Fig 7:**
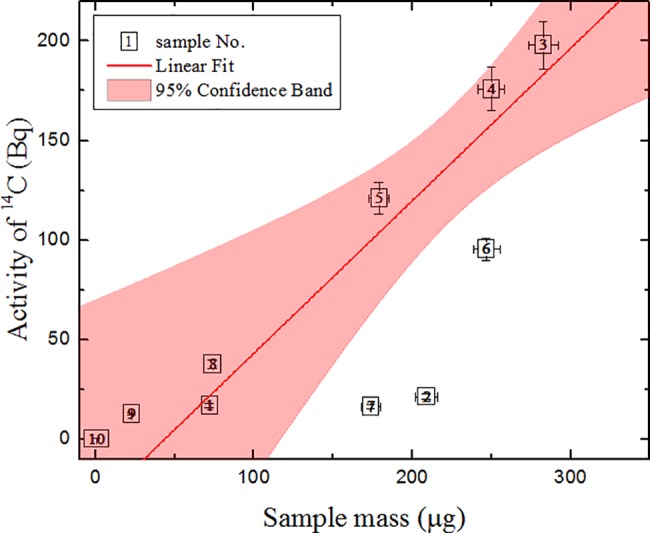
Correlation between ^14^C activity and the mass of graphite samples. The parameters of linear approximation (y = a + bx) are: a = -34 ± 45, b = 0.8 ± 0.2; R = 0.62. Sample mass was determined by the elemental analyzer.

The correlation between the ^14^C activity and mass of graphite samples is fairly good, with a correlation coefficient of ~0.62 (see Fig 7). Samples No. 2, 6–7 may be outside the 95% confidence band because of the slightly different structure of the material (density variation) or inhomogeneous distribution of its impurities.

**Fig 8:**
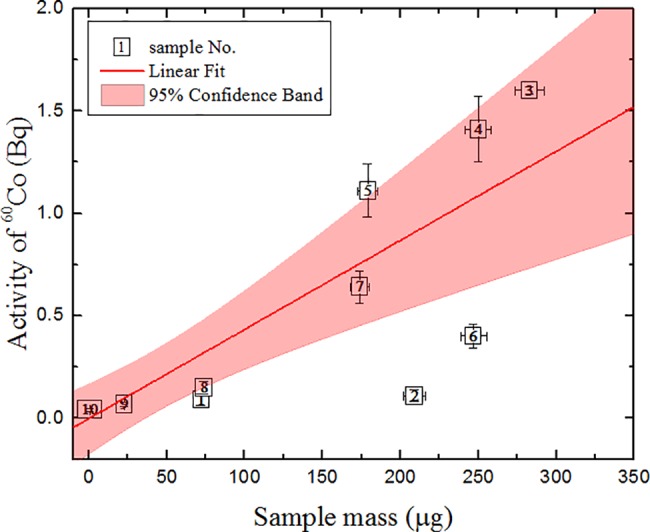
Correlation between ^60^Co activity and the mass of graphite samples. The parameters of linear approximation (y = a + bx) are: a = –0.004 ± 0.07, b = 0.004 ± 0.001; R = 0.74. Sample mass was determined by an elemental analyzer.

The same finding is valid for the correlation between ^60^Co activity and the sample mass (R = 0.74 see Fig 8). This means that most of the heavier samples with higher ^60^Co activity would also have higher ^14^C activity. The deviation due to sample structure peculiarities is also observed in both ^14^C and ^60^Co activity correlations with the sample mass, as was the case in samples No. 6 and No. 2.

In some cases, ^137^Cs gamma measurements may also serve as a key for the determination of other nuclides (from uranium impurities in the graphite). But in this experiment, ^137^Cs activities are either very low compared to ^60^Co activities or below detection limits in the measured samples and no correlation with the sample mass was observed.

**Fig 9:**
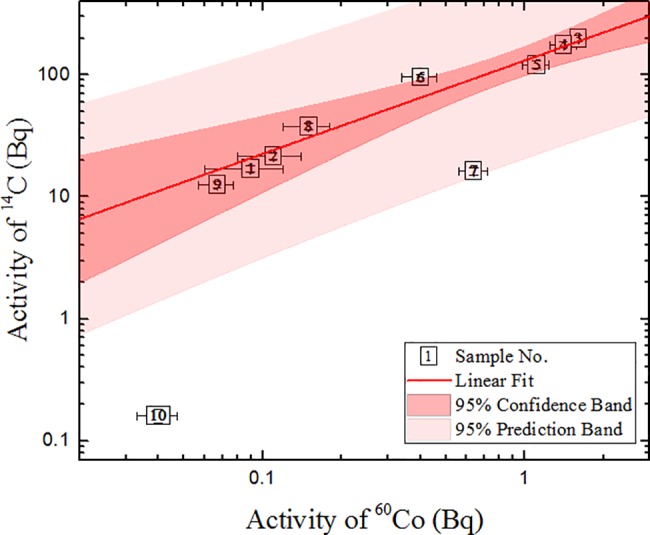
Correlation between ^14^C and ^60^Co activities of the graphite samples. The parameters of linear approximation in the logarithmical scale (lg(*Y*) = *a* + *b*· lg(*X*)) are: a = 2.11 ± 0.05, b = 0.8 ± 0.1; R = 0.77.

The ^14^C/^60^Co ratio of activities in the graphite samples is also listed in Table 1. These ratios are different in each particular sample. This is attributed to the different concentration of impurity elements and the different activation reaction cross sections in the particular neutron flux. This ratio is also important in determining the ranges of the sample impurity concentration variation, when divided parts of the sample are compared. For all samples, except No. 10, a correlation between ^14^C and ^60^Co is observed: the correlation coefficient is ~0.77 (see Fig 9 for details). However, the number of samples is not sufficient to conclude that ^60^Co could be used as a key nuclide in graphite ^14^C scaling factor determination.

### ^14^C activity measurements in graphite using semiconductor detectors

During one combustion cycle, not all the graphite present in the tin capsule is burned down immediately. Additional combustion cycles were needed to burn down the graphite sample completely. The final combustion cycle is determined when there is a cycle in which the TCD in the elemental analyzer shows no CO_2_ signal—meaning all carbon from the sample was burned down in the previous combustion cycles. All counts above the background are accounted during the online activity determination. The total activity of the sample is the sum of the pulses from all combustion cycles of one sample. For the LSC measurements, gas catchers are not changed between cycles and they are used until all carbon from the sample is burned down and trapped in the solution.

The time needed for complete graphite sample combustion may depend on the sample size and the amount of oxidant added. The reason for variation in the amount cycles needed for the full combustion of the sample can be associated with the structure of the graphite sample. A more porous sample with a larger specific surface area would completely burn up in a shorter period of time compared to a compact graphite sample.

As previously described in this study, ^14^C activity of a particular graphite sample was determined with semiconductor Si detectors. In order to perform online ^14^C activity measurements, the detector chamber with two Si detectors was located between the sample combustion system and CO_2_ alkali catchers. Additionally, LSC measurements were carried out after completed sample combustion and CO_2_ collection by trapping in NaOH solution.

**Fig 10:**
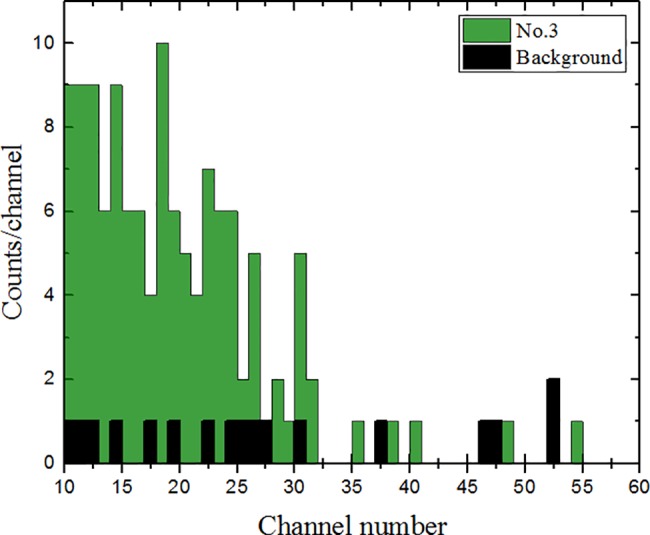
An example spectrum of ^14^C measured using semiconductor detectors. The green color denotes the spectrum of sample No. 3 and the black color denotes a typical background spectrum. Both spectra were collected over 100 s time intervals.

The spectrum of ^14^C measured using semiconductor detectors during the combustion of sample No. 3 is presented in Fig 10. The presented spectrum was measured between 200 and 300 seconds of the first combustion cycle (when CO_2_ gas was evolving from the elemental analyzer).

**Fig 11:**
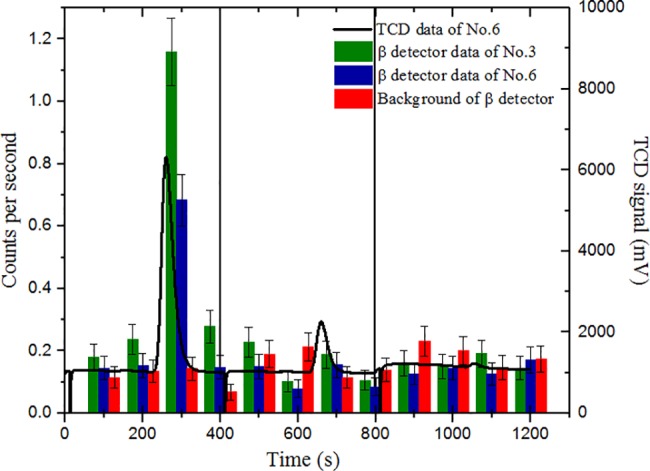
CO_2_ flow intensity signal measured by TCD (black line) and cps spectra of samples No. 3 (green), No. 6 (blue) and background (red) as determined with the semiconductor detector. In total, three combustion cycles (400 s each) were used to completely combust the non-ground graphite sample.

In the used configuration, CO2 gas comes to the semiconductor detection system over a period of 200–300 seconds within the first combustion cycle. Additional subsequent combustion cycles with the same timing were used to completely burn down graphite until the TCD would not show the CO2 flow and β detection system detected no significant changes between measured and background spectra. Combined spectra of TCD and the β detection system are shown in Fig 11. This type of distribution is seen in all the samples and it is due to the strong decrease in the fraction of CO_2_ gas in the later cycles.

**Fig 12:**
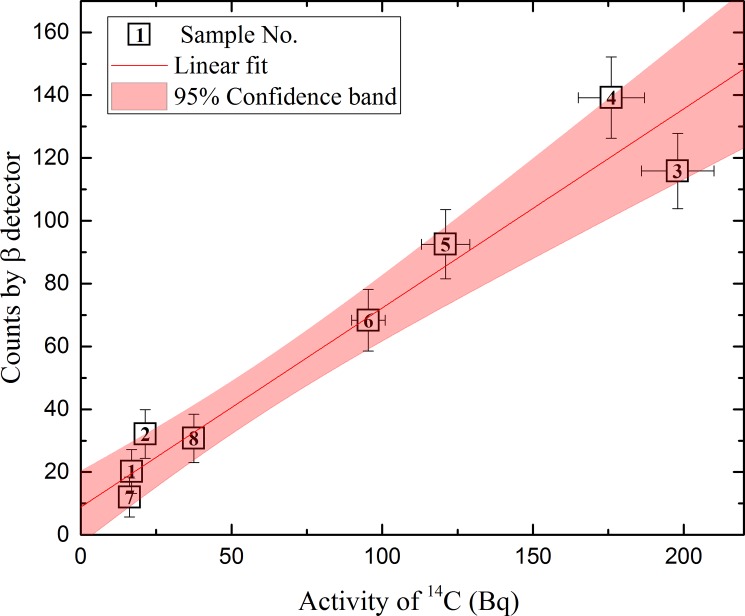
Correlation between LSC and semiconductor detector data. The horizontal axis represents LSC measurement data of ^14^C activity and the vertical axis represents semiconductor β detector pulse counts obtained per 100 s. The parameters of linear approximation (y = a + bx) are a = 8.83, b = 0.63, R = 0.94.

Fig 12 represents the correlation between LSC and semiconductor detector measurements. A linear dependence is observed in the measurements of both methods.

Semiconductor detector count data were taken only from the third step of the first combustion cycle (within 200 and 300 seconds). Considering the data from Fig 10, it could be assumed that most of the radioactive nuclides flowed through the system in the period spanning 200–300 seconds. Errors in the linear correlation could appear due to the residual activity in gas, as gas flows inside the detector chamber. However, the confidence band of the linear approximation function shows that the uncertainty in the correlation between the two methods of ^14^C activity determination is in the range of 10–20%. This is a reasonable range for radioactive waste characterization purposes.

## Discussion

Quantifying the specific activity of radionuclides in radioactive waste is particularly relevant when assessing radioactivity to determine the appropriate treatment, packing, storage and final disposal methods. According to the existing radiological classification, irradiated graphite waste is classed as long-lived low and intermediate activity radioactive waste [25]. As ^14^C dominates the activity of radioactive graphite waste (about 40% for the first 30 years and almost all of the activity for the rest of the time), it is important to develop less costly and less time consuming experimental means for measuring the specific activity of this β nuclide. This is particularly relevant when measuring ^14^C activity in irradiated graphite from the nuclear power plants, during reactor dismantling.

^14^C is a β emitter with relatively low-energy of electrons (E_βmax_(^14^C) = 156.5 keV). In order to determine ^14^C activity several direct counting techniques can be applied: ^14^C can be detected using an ionizing chamber, a gas flow proportional counter or Geiger–Müller (G-M) detectors. However, β emitters should be either inserted directly into the active chamber filled with ionizing gas, or detectors should be windowless or have a thin-window (for example 1.5–4 mg/cm^2^) sheet of mica or Mylar, which allows a minimum distance to the sample. The electronic signals from the passage of individual particles through an ion chamber can be accurately measured, along with the energy deposited in the gas as a function of position inside the volume. Recently, thermal oxidation combined with the gas detector technique has been used to determine ^14^C concentration in irradiated graphite from an Oldbury reactor [10]. Detectors that are operating in the proportional region with 4π registration geometry systems and ultra-thin windows have ^14^C detection efficiencies that are typically up to 40% [26]. Appropriately configured G-M detectors are more sensitive to X-rays, γ-rays and high energy β particles. Although being less sensitive to low energy β particles, as emitted by ^14^C, these detectors can be designed for β radiation detection as end-window or pancake tubes depending on the window thickness. One such example is the multi low level counter FHT 770T (Thermo Fisher, USA) which offers the measurement of contamination down to the mBq range with a relatively short measurement time with a typical detector efficiency of 30%. Because it is sensitive to all β emitters, it could not be used for radioactive graphite ^14^C characterization as it would detect the sum of β radiation, and would also require a different sample preparation procedure.

An alternative to the aforementioned methods is LSC—the most sensitive technique (with an efficiency of 85%) for the sophisticated determination of ^14^C [27]. LSC is an analytical technique which uses liquid chemical medium capable of converting the kinetic energy of nuclear emissions into emitted photons. Liquid scintillation counters are ideal for counting radionuclides that decay by emitting β particles (for example: ^3^H, ^14^C, ^35^S, ^36^Cl, ^45^Ca).

Semiconductor detectors are around 10^3^ times denser than gases and have lower ionization potentials, thus they are more sensitive than gas detectors and after thermal oxidation of the sample, they could be used as spectrometers [28]. However, not all of the active volume is used for radionuclide determination in the gaseous form. The registration efficiency can reach as high as 100% when a large volume of detection material is used. The achievable efficiency is less than 50% in ideal conditions when 2 planar detectors in a 2π geometry are used.

As the aim of this work is to demonstrate the feasibility and effectiveness of the proposed rapid analysis method for ^14^C specific activity determination, we investigated small, cylindrical graphite samples from the Ignalina NPP. ^14^C is generated by neutron activation predominantly in ^13^C(n, γ)^14^C,^14^N(n, p)^14^C reactions. Before this ^14^C activity examination, gamma spectroscopic analysis was performed for each sample to determine the gamma emitters ^60^Co and ^137^Cs. This was done to determine if there was a possible correlation between ^14^C and easily measureable radionuclides. After this, gamma spectroscopic analysis samples were broken into smaller pieces for combustion.

In this work, we demonstrate a rapid analysis method for determining ^14^C specific activity in small (<100 μg) irradiated graphite samples from a nuclear power plant. The carbon mass in the sample was measured simultaneously with an elemental analyzer after the graphite combustion process. ^14^C activity in the released CO_2_ gas was measured online with a convenient semiconductor detector or, later, by the LSC method.

In order to determine the ^14^C specific activity, the sample must first be weighed and this could be problematic due to higher measurement uncertainties or in case where there are a large number of measured samples. The proposed method allows one to determine the ^14^C activity in the samples, without needing to define the carbon mass as carbon quantification is performed in the elemental analyzer. Using the proposed configuration, we were able to determine the ^14^C specific activity of graphite samples which contained less than 1 μg of carbon—particularly significant taking into account the high activities of radioactive waste generated by the nuclear reactor.

The experiments showed that the proposed method could be applied as an online measurement system for determining ^14^C activity. The correlation between the graphite sample mass obtained by weighing, and the elemental analyzer results after combustion indicates that the carbon mass in the sample is determined with sufficient accuracy (the correlation coefficient is equal to 0.97, see Fig 6). It must be mentioned that samples as small as 1 μg can be measured, which makes this method especially favorable for small samples. By using the elemental analyzer and the detector chamber with two semiconductor detectors, the ^14^C specific activity in samples containing more than 19 Bq of ^14^C can be determined in less than ten minutes. By comparison, the more complicated and time consuming LSC method is also sufficiently accurate for the same activity level (a statistical uncertainty of 1% is achievable within 12 minutes of counting the samples). However, LSC sample preparation takes much longer (up to 5 hours). The limitation of 19 Bq appears from the background data analysis: the analysis of 80 data sets showed that a mean is 23.2 and a standard deviation (SD) of background data is 5.4. The statistically reliable activity of the detection could be described by value of 3 SD, which is 16 counts per 100 seconds. 16 counts per 100 s correspond to the gas activity equal to 19 Bq. In the cases of the samples with lower total activities (as No 1 and 7) the values of measured signals were higher than 2 SD compared to background and additionally they were measured with LSC. Because of that they were considered as sufficiently reliable ones for analysis. But taking into account the common background the method is validated for 19 Bq activity of the sample. In the case of lower activity samples or when the additional checking of specific samples is required, the CO_2_ gas can then be trapped after measurement with our method, and measured using LSC as well. Good correlation between the results of the semiconductor detector and LSC was obtained in the range of 19–200 Bq. Semiconductor detectors can be used for rapid ^14^C activity determination, while if higher accuracy of ^14^C measurement is needed, the LSC method should be used additionally, as the latter is a well-established and widely used method for ^14^C activity determination.

The linear approximation function (between the activity measurements from the LSC method and the semiconductor detectors) that is presented in Fig 12 could serve as the ^14^C activity calibration curve and could be used for rapid ^14^C activity determination in routine measurements. After a complete procedure of graphite measurements (LSC measurement for every 20^th^ semiconductor detector measurement) one could easily perform sorting of certain graphite constructions and even obtain the nuclide vector based on ^14^C, when combining both ^14^C and ^60^Co measurements for other difficult-to-measure nuclides that are important to graphite treatment and disposal.

The proposed method can also be used in biomedical applications, where the ^14^C specific activity needs to be analyzed in small bioorganic samples. While ^14^C measurement in of itself can be performed with any detection system, determination of the carbon content for ^14^C specific activity evaluation in the sample can be problematic due to the presence of additional substances. In our proposed concept, the carbon amount is determined without sample preparation, while the ^14^C amount in the sample is measured online or later using the LSC method. Using the online configuration, the specific activity of the bioorganic sample can be assessed in less than ten minutes, which makes it favorable for biomedical applications.

## Conclusions

^14^C is the major contributor of activity in radioactive graphite waste and it is important to measure its specific activity, in a reasonable time and at a relevant cost, during the dismantling of nuclear power plants.

The rapid analysis method for ^14^C specific activity determination described here has shown good results and could be used to examine irradiated graphite. The rapid analysis method is based on graphite sample combustion and total CO_2_ mass evaluation using a commercial elemental analyzer and subsequent ^14^C specific activity determination using a semiconductor detectors system. Samples as small as 1 μg can be measured, which makes this method especially favorable for small samples, whose activities could be measured either with a liquid scintillator or with a semiconductor system, depending on the target. The method using semiconductor detectors was cross-checked using LSC measurements and gas catchers (3 M NaOH) to evaluate the accuracy of the measurements for very small mass samples with low ^14^C activity. The linear approximation function between the activity as measured by the LSC method, and as measured with semiconductor detectors could serve as the ^14^C activity calibration curve and could be used for rapid ^14^C activity determination in routine measurements. The important feature of this method is the fast analysis of the specific activity without a separate sample weighing procedure: the detection procedure for samples containing a ^14^C activity higher than 19 Bq takes approximately 10 minutes, compared to the more complicated and time consuming LSC method. Notwithstanding that the estimated efficiency of the semiconductor detectors system is fairly poor (15%), due to the less than the ideal 2π geometry, the uncertainty of the rapid method is within an acceptable range (10–20%) for radioactive waste characterization purposes. Due to the fast, online measurement, and ^14^C specific activity evaluation, this method can be applied to online radiological characterization during the dismantling of reactor graphite constructions, as well as applied to the sorting of graphite waste. The conventional and accurate LSC method could be used in control measurements (for example, every 20^th^ sample) of selected/specific samples during the characterization procedure. The proposed rapid analysis method allows far more samples to be analyzed in an automated and timely manner. This method could be used to directly characterize radioactive waste using a scaling factor method or even used in biomedical applications when dealing with the specific activity determination of ^14^C in a sample.
